# Vector Potential of *Nosema*-Infected Drones in Honey Bees

**DOI:** 10.3390/insects16111142

**Published:** 2025-11-07

**Authors:** Adrian Perez, Brian R. Johnson

**Affiliations:** Department of Entomology and Nematology, University of California, Davis, 1 Shields Avenue, Davis, CA 95616, USA; arez@ucdavis.edu

**Keywords:** social immunity, honey bee, *Nosema*, social networks, beekeeping

## Abstract

Despite the advantages associated with social living, one of the main costs of sociality is an increased exposure to pathogens. For honey bees, these costs are artificially inflated in the unnatural and densely crowded apiary environments common to commercial operations. Modern beekeeping practices have not only exacerbated these problems but have also led to the introduction of novel parasites. One newly introduced parasite of honey bees is *Nosema ceranae*, a unicellular, spore-forming gut parasite that has achieved a cosmopolitan distribution in recent decades. *N. ceranae* is widely considered a dangerous pathogen for both individual bees and colonies as a whole. The parasite can infect every member of the honey bee colony; however, it remains to be understood whether there are asymmetrical contributions to spreading *N. ceranae* spores by particular castes. Here, we test the long-held suspicion that honey bee drones may be important for driving pathogen transmission between colonies in modern apiary settings. Although we find sparse evidence that *N. ceranae* infection causes enhanced transmission by changing drone or worker behavior, we do confirm that infected drones drift at high rates and are accepted by foreign colonies. Our results thus present a grim picture for enhanced diseases transmission by males in crowded apiaries.

## Simple Summary

Group-living confers many advantages; however, there are also costs, the chief of which is that crowded nests can greatly increase the rate of disease transmission. The problem is particularly acute for managed social animals like the honey bee because many nests are crowded into one place. Most research on honey bees focuses on the workers and queens, the females, while little attention is given to the males, called drones. However, drones are thought to be the most likely to drift between hives in an apiary, suggesting they may be important vectors of disease. Here, we test a series of hypotheses suggesting that drones may spread a key fungal pathogen, *Nosema ceranae*. We find that infection with this fungus does not change drone behavior; however, infected drones are readily accepted into foreign hives because of drifting, which we confirm is pervasive for honey bee males. Infected foreign drones engage in food exchange with their new hosts and move about the nest providing ample opportunity for disease transmission. Our work suggests further attention should be given to the neglected drone with respect to their role in the spread of pathogens and parasites.

## 1. Introduction

Western honey bees (*Apis mellifera*) are an important pollinator species that produces billions of dollars in agricultural production in the United States and around the world [1,2]. In recent decades, there has been much concern over the declining health of economically important insect species that are struggling with both longstanding and novel challenges in a rapidly changing world [3,4]. Along with factors such as pesticides and poor nutrition, a key component determining the overall health of honey bees is their ability to withstand myriad pathogenic pressures [5]. Honey bees live in large societies composed of closely related individuals housed in densely populated, temperature-controlled nests. These characteristics, along with frequent social interactions between nestmates, make these bees especially attractive targets for a variety of fungal, viral, bacterial, protozoan, and ectoparasitic agents [5,6].

In association with the diversity of pathogens that honey bees can host, there are numerous sources of infection and modes of transmission that can initiate and spread disease outbreaks in colonies. Most previous work on this topic has highlighted the role of foraging workers, which leave the nest and potentially pick up pathogens in contaminated nectar, pollen, or water [7,8,9,10]. Honey bee drones, the male reproductives of the colony, have long been speculated as reservoirs and vectors of harmful pathogens [11,12,13]. However, the actual epidemiological impact of drones has mostly been narrowly focused on the context of venereal and vertical transmission to a queen and her future offspring [14,15,16,17,18].

There are robust reasons to expect that drones may have a sizable impact on disease dynamics beyond pathogen transmission during mating. Developing drones are preferentially parasitized by Varroa mites and can emerge carrying viruses vectored by the mite [19,20]. Drones at all life stages are also commonly infected with fungal pathogens such as *Nosema* species [12,21]. Drones have also been shown to exhibit lower immunocompetence than workers at all developmental stages [22]. With respect to horizontal transmission between colonies, drones are known to fly into neighboring colonies (i.e., “drift”) up to three times more than workers [12,23]. It is therefore recognized that drones host notable pathogens, may be highly susceptible to infection, could exhibit larger pathogen loads than workers, and are capable of introducing pathogens into nearby colonies, especially in crowded commercial apiaries [21,23].

*Nosema ceranae* is a microsporidian pathogen that drones could play a role in spreading across colonies in commercial apiaries [21]. *N. ceranae* is an obligate parasite of the honey bee midgut that produces spores to propagate itself between hosts mainly via fecal-oral and oral-oral routes [24,25]. While the original host of *N. ceranae* has not yet been definitively determined, the current understanding of its recent spread and distribution are consistent with the hypothesis that its original host is the Eastern honey bee *Apis cerana* [26,27,28]. Hence, researchers have operated with consensus that *N. ceranae* is an evolutionarily novel parasite for *A. mellifera*, the honey bee species most commonly used for pollination [28]. The exact virulence of *N. ceranae* has been inconsistently determined across geographic regions [29,30,31,32]. Still, it is clear that the pathogen has negative effects on individual bees that could overwhelm a colony with high levels of infection [33,34,35,36].

The effects of *N. ceranae* on *A. mellifera* have received considerable attention in the female worker caste of honey bees. Previous research has identified a wide array of symptoms such as increased hunger, decreased carbohydrate and lipid levels, suppression of immune responses, decreased flight ability, decreased navigation and homing success, and increased preference for warm temperatures [37,38,39,40,41]. Although host manipulation by a pathogen is difficult to prove [42,43], some of these behavioral and physiological changes could positively impact the spread of *N. ceranae*. Decreased navigation ability could lead to more drift, which in tandem with the suppression of immune responses that are known to trigger aggressive encounters towards immune-challenged bees, may create greater success for sick bees attempting to enter neighboring hives, as guards may be primed to pay great attention to bees with activated immune systems [44,45]. Combined with increased hunger, infected individuals could pass on spores readily as they beg to be fed by workers in colonies they drift into [24]. As for the special importance of drones, caged experiments comparing transmission rates of *N. ceranae* from either infected drones or infected workers to naïve workers have shown greater rates of transmission in cages where drones served as the source of infection [46].

Here, we explore the effect of *N. ceranae* infection on honey bee drones across multiple contexts to assess their potential impact on transmission dynamics within and between colonies. Our hypotheses for each experiment generally suppose that colonies attempt to defend themselves against *N. ceranae* infection through common social immunity mechanisms (e.g., avoidance of sick individuals, [6]). However, we also recognize that *A. mellifera* may have not had sufficient time to evolve sophisticated strategies against this relatively novel parasite [47]. We test the effects of *N. ceranae* infection on: (1) the drift rates of drones and their space use within colonies, (2) the ability for drones to enter novel colonies undetected, (3) the social interactions drones successfully initiate in novel colonies, and (4) the energetic stress experienced by drones that may underlie pathogen spread via social feeding.

## 2. Materials and Methods

### 2.1. Overview of Experimental Approaches

We begin with a brief overview of the basic design and intention of each experiment we used to test distinct aspects of the vector potential of *Nosema* infected drones. First, we present an experiment validating that our particular method for inoculating large groups of drones produces a sufficient prevalence of infection in our treatment drones that exceeds the background rate of infection in our control drones. This experiment underpins our confidence that the results seen in our later studies can be attributed to *Nosema* infection. Second, we present an experiment to measure the drifting rates of drones in field colonies as well as how their spatial preference for central areas of the nest changes over time. This experiment addresses the ability for infected drones to enter novel colonies in an apiary and offers some information on which members of the colony may be interacting with infected drones based on where they spend time in the nest. We then describe a laboratory assay used to directly test if infected drones receive more aggressive or affiliative responses from putative guard bees in a small arena. This assay provides insight into the potential for infected drones to experience increased or decreased success during drift events regardless of if they actually attempt to drift more or less than uninfected drones. The fourth experiment focuses on the social feeding interactions of infected and uninfected drones when introduced to a novel colony. This experiment allows us to confirm whether or not workers in a colony will readily interact with foreign (i.e., drifted) drones and if infection has any positive or negative effect on interaction rates. Finally, the last experiment tests the food consumption of infected drones in caged laboratory trials. This experiment aims to reveal large changes in appetite due to infection with a gut pathogen that may then lead to increased propensity to seek social feeding and therefore increase pathogen transmission.

### 2.2. Group Inoculation

We inoculated drones in groups which have been reported to produce high rates of infection in workers [48]. In general, we made several adjustments to standard protocols due to the unique challenges of working with drones and the large number of individuals used over the course of multiple experiments. To verify the efficacy of group inoculation for drones, we tested the prevalence and intensity of infection in inoculated drones and drones given untreated sugar solution in cages at four-, six-, and eight-days post-inoculation. These times mirror when drones were used in later studies. We performed this experiment with both “newly emerged” drones and “mature” captured free flying drones, both of which were used in later experiments, although not mixed together. Inoculation experiments were performed on mature and newly emerged drones in separate trials, and each of the subsequent experiments only used one of these types of drones for their trials. We used one cage of drones per experimental combination per trial (e.g., one Control-4 days-Mature cage, one Treatment-4 days-Newly Emerged cage, etc.). We performed this experiment with mature and newly emerged drones twice each and pooled the data from both trials together for each exact cage combination.

Prior to introducing drones to inoculation cages (wood with a glass front panel; 15 cm × 10 cm × 8 cm), we added nurse bees to each cage by brushing in workers from open brood frames from a randomly selected field colony. Each cage received enough nurse bees to maintain about a 1:1 ratio of nurses and drones. We then collected drones from the top hive box of three to five strong field colonies as adults (“mature” drones) or retrieved them from frames of emerging drone brood left in an incubator at 35 °C for two days to allow for a sufficiently large batch of drones to emerge (“newly emerged” drones). Drones were then added to each cage in groups of 50 and the cage was given either unadulterated sucrose solution (50/50 wt/wt) or sucrose solution with fresh *N. ceranae* spores. The inoculate primarily contained *N. ceranae* with potentially small amounts of *Nosema apis.* We were confident that our inoculations would produce *N. ceranae* infections because *N. ceranae* has largely outcompeted *N. apis* in our geographic region and because *N. ceranae* is present in colonies year-round while *N. apis* is known to be eliminated from colonies during warmer months [21,28,49,50,51,52,53].

We prepared inocula by capturing bees from the entrance of multiple field colonies using an aspirator. We collected workers from the hive entrance and lid to primarily capture older bees. Collected bees were then put into a refrigerator (3 °C) until rendered immobile. We dissected the immobilized bees for spores in groups of 25–50. We removed the abdomen of each bee, put the abdomens in a pestle with one mL of water per bee, and ground the contents into a slurry. The slurry was then filtered through a Kimtech Wipe and a piece of Whatman #4 filter paper. We then quantified the number of spores in each mL of solution using a Neubauer^®^ hemacytometer (LW Scientific, Lawrenceville, GA, USA) as per [54]. We mixed the appropriate amount of slurry to form a sucrose solution containing about 500,000 spores per bee. 500,000 spores per bee is much higher than the reported ID100 for *N. ceranae* but was used to compensate for the need to have nurses feed on the inoculate and pass spores on to drones. Nurses and drones were housed in an incubator at 35 °C for 48 h to allow time for nurses to feed and inoculate the drones. After 48 h, both groups were fed untreated sucrose solution ad libitum.

At the appropriate time points, we randomly selected 20 live drones from each cage and sacrificed and stored them for later dissection. To achieve the prevalence and intensity of infection, we removed the abdomens of drones individually and macerated and filtered the abdomen slurry as we did when making the inoculate. Since drone gut contents have more unwanted material that makes it through the filtering stage, we used 2 mL of water per abdomen and adjusted for extra dilution in the spore count calculation [54]. We calculated the prevalence of infection as the proportion of drones in each cage that presented with spores and the intensity of infection for each individual was their spore count estimate. All analyses, for this and later experiments, were performed in R version 4.3.3 and R studio [55]. To test if inoculation significantly affects the rate of infection in our cages, we used a chi-square test. To compare the distributions of spore counts between groups we used Mann–Whitney U tests.

### 2.3. Drifting and Space Use

For this experiment, we introduced marked drones to a focal field colony and routinely inspected the focal colony and two neighboring colonies to assess the rate of drifting of drones and the spatial location of drones in the host and novel colonies. This experiment consisted of three trials performed at separate field sites over a three-month period, July to September 2022. Each trial began by moving three field colonies to a secluded area of a new apiary with hives placed in a straight line and given 1 m of space between hive entrances. All colonies were initially standardized for strength and frames were replaced and shifted as necessary to establish a discrete center brood zone and outer food zone in each colony [56]. The center brood zone consisted of five frames in the middle of each hive box filled predominately with open brood and some closed brood. The food zone consisted of the remaining two or three frames on each side of the brood zone frames, with these frames predominately filled with open nectar cells, some capped honey, and some empty cells. Colonies were given a minimum of two weeks to acclimate before use in the experiment.

We obtained drones from frames of emerging drone brood in colonies not used in the experiment trials. Frames were housed in an incubator in a five-frame plastic nucleus box. After two days, the newly emerged drones were transferred to a plastic bin to be paint-marked on their abdomen with a color corresponding to whether they would be inoculated for the trial. Once marked, the drones were transferred to housing cages in groups of about 40. We inoculated newly emerged drones with our standard group inoculation procedure and introduced both inoculated and control drones together to the focal field colony at four days post-inoculation so that drones could develop infections over the course of the experiment without high mortality early in the trial. The middle colony served as the host colony in every trial. Drones were introduced to the top hive box of the host colony at midday. In preparation for the drones to be introduced, we added a queen excluder panel between the top and bottom hive box, as well as below the lid, to facilitate acceptance in the colony as drones could not walk out or be forcibly removed from the colony. Interestingly, only voluntary self-removal was seen in preliminary trials, but this was observed for both inoculated and control drones. 48 h later we removed the queen excluders, and the drones were free to exit the colony and begin flying.

Inspections started the morning after the queen excluders were removed and occurred every three days afterwards. The maximum time allotted for each trial was 19 days with seven inspections total. Drones were 23 to 25 days old at the end of the trials. Inspections were conducted as follows: starting with the top super, all frames were removed one at a time with pictures taken of both sides of each frame. The same process was repeated for the lower hive body. We then reassembled the colony with frames placed back in their original positions. Inspection images were later examined for the presence of drones. For each spotted drone, we noted the colony the drone was found in (host or novel), the color mark on their abdomen (inoculated or control), and the frame type on which they were located (brood or food). Frame type was determined by the majority of cell contents with food and empty frames grouped together as “food” frames for analysis. “Brood” frames represented the central frames that predominately consisted of open or closed brood cells. Analyzing each trial separately, we tested for the effects of treatment and time on drone drifting and space use using binomial generalized linear models with inoculation and day of inspection as fixed effects. Drift and space use were both coded as binary outcomes for this analysis (i.e., counts of drift and usage of the brood zone were coded as “successes”).

### 2.4. Intruder Assay

To explore the responses of workers at the colony entrance to a novel drone we used intruder assays. Intruder assays are a common controlled laboratory approach for studying aggression events [57]. Assays were performed in the summer of 2021. Due to issues with the availability of newly emerging drones, we used mature adult drones captured from three to five colonies with high drone production. Drones in the top hive box of these colonies were handpicked and put in holding cages for transport. The ages of the drones are therefore unknown, but collected drones were randomly assigned to the control or inoculated cages. We then inoculated drones as in the drift study but maintained the cages until 6 days post-inoculation so that infections were more developed during the single assay in which drones were tested. We performed four rounds of trials and gathered data on 54 treatment drones and 61 control drones.

We used an aspirator to collect workers from the entrance of a single source colony in order to gather putative guard bees and foragers to populate the assay arenas. This method ensures all workers are from the same colony and are also older, more aggressive guard bees or foragers. These workers were chilled until immobile and then distributed into assay arenas in groups of six. Workers were given at least 10 min to regain mobility and acclimate to the arena. Arenas consisted of a single piece of wax foundation (Mann Lake) cut to fit a 100 mm Petri dish. We then assayed each drone once in 10 min filmed trials. Each group of workers was only used for a single assay. Arenas were reused across rounds of trials but were sanitized in a freezer in between trials [32].

We scan sampled each video every 30 s for behaviors received by the focal drone from workers: grooming, antennation, trophallaxis, and aggression (an aggregate of stinging, biting, and chasing). We also made note of one drone behavior, food begging (extension of the proboscis out towards the mouth of a worker), as this behavior may indicate that treatment drones are experiencing more energetic stress in comparison to control drones. We analyzed the results for each behavior separately with binomial generalized linear mixed models using inoculation status as a fixed effect and trial as a random effect.

### 2.5. Social Interactions

The goal of this experiment was to characterize the interactions between foreign drones that had entered a new colony and the workers in the novel nest with particular attention paid to which members of the colony are interacting with sick or healthy foreign drones. In other words, in a situation mimicking the drift of drones into a novel colony, which workers are willing to interact and share food with these foreign drones? We performed this experiment in two trials in August and September 2021 in separate observation hives with populations sourced from various field colonies that were not used for drone collection. To establish a colony of 1500 workers identifiable by age, we introduced 300 newly emerged workers in weekly cohorts over five weeks into a two-frame observation hive. Each cohort was painted with a different color applied to the abdomen to identify their age. Marked workers were between 4 and 32 days old at the trial.

We captured and inoculated mature drones as in aforementioned experiments. Additionally, drones were marked with paint applied to the abdomen prior to being split into control and treatment cages for group-level identification. Drones were kept until 6 days post-inoculation before introduction to the focal observation hive. On the day of the experiment, we switched the bottom frame of the observation hive to the top of the hive and added a queen excluder between the top and bottom frames. This manipulation eliminated the option for drones to leave the colony, either through their own choice or through ejection. Drones were observed leaving the observation hive of their own accord in preliminary trials of the experiment; however, no apparent aggressive interactions were ever witnessed. Immediately after introduction, we took one hour of footage of both sides of the top frame of the observation hive.

We manually collected data from each video focusing on mouth-to-mouth food sharing events. For each interaction, we noted the identity of the drone as either inoculated or control, the age of the nectar donator, and the duration of the event. We tested for differences in the distributions of trophallactic durations with each cohort using Mann–Whitney U tests.

### 2.6. Energetic Stress

To directly assess the potential energetic stress caused by *Nosema* infection, we employed a simple test of hunger using feeders weighed before and after daily syrup consumption by drones kept in cages. While this experiment has been performed with inoculated workers in the past using gravity feeders, we found in preliminary testing that drones cannot readily learn to use these types of feeders and instead tend to starve regardless of infection. To circumvent this issue, we used cut sections of wax comb (~6 cm × 6 cm) that we filled with sucrose solution and placed at the bottom of cages for drones to directly feed from as they would in a normal colony. We performed two trials of this experiment during the summer of 2021 using five treatment cages and four control cages in trial 1 and six treatment and control cages in trial 2.

We captured and inoculated mature drones as in previous experiments with the only distinction being that drones were introduced into experimental cages at three days post-inoculation. Drones were distributed into cages in groups of six to eight, given an initial weighed comb piece with food, and housed in an incubator for 24 h. Each day after old comb feeders were taken out of cages and weighed, new comb feeders were filled with food, weighed, and introduced to the cage; and dead drones were removed and counted for survivorship data. We executed this procedure for six days before ending each trial.

Using the change in weight between days and survival data, we calculated the average consumption of food per living drone for each cage during each day of the experiment. Because open comb loses weight in the incubator due to evaporation alone, we corrected each weight change measurement with the average daily weight loss in a set of 21 comb sections used in the experiment and left in the incubator for 24 h without any exposure to bees. This average value was subtracted from each experimental value, and the resulting corrected value was used for analysis. Negative values in cages with alive drones were kept for analysis; however, negative values in cages where all drones were dead at the next morning inspection were not used for analysis. In the first case, it was assumed the drones ate a negligible amount of food over a single day but were alive and would eat the next day; while in the latter case, we assumed the drones all died early in the day before being able to consume a noticeable amount of food and so the data were excluded. All values were adjusted by adding the absolute value of the largest negative value in each trial to each measurement and then square root transformed prior to analysis. We analyzed per drone food consumption in each round of trials separately using linear mixed models with infection and day as fixed effects and cage ID as a random effect.

## 3. Results

### 3.1. Group Inoculation

**Figure 1:**
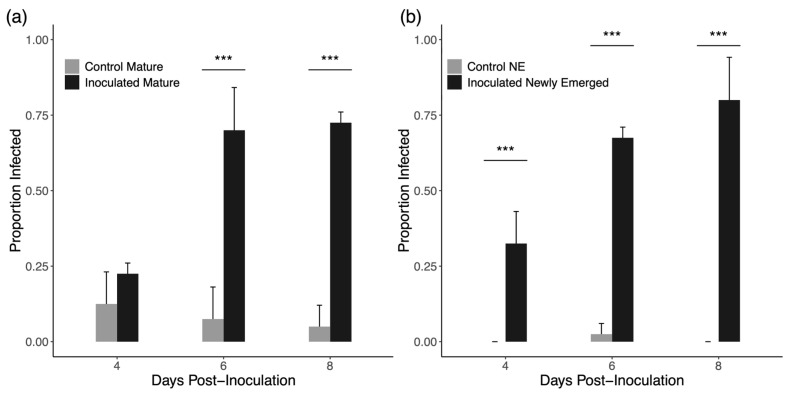
Average proportion of drones presenting with spores in each group +1 standard error. (**a**) Group inoculated mature drones show higher rates of infection at 6 days and 8 days post-inoculation but not 4 days post-inoculation. (**b**) Group inoculated newly emerged drones show higher rates of infection at all time points post-inoculation. *** denotes a significant difference.

Across the two trials, we obtained data on 40 drones for each cage combination (mature or newly emerged; inoculated or control; four-, six-, or eight-days post-introduction). Figure 1 shows the average rates of infection in mature and newly emerged drones. Rates of infection in inoculated mature drones ranged from 25 to 80% with much higher rates in both the six- and eight-day groups. For uninoculated mature drones, infection rate was highest in the four-day group, but spores were observed in a few control drones at all time points. Newly emerged drones show a similar pattern overall, but it is worth noting that uninoculated newly emerged drones very rarely presented with spores (just one drone in a six-day cage). For mature drones, inoculated drones had a higher rate of infection six- and eight-days post-inoculation (χ^2^ = 30.336 for six days and χ^2^ = 35.602 for eight days, *p* < 0.0001 for both time points) but not at 4 days post-inoculation (χ^2^ = 0.779, *p* = 0.377). For newly emerged drones, inoculated drones had a higher rate of infection at every time point (χ^2^ = 13.226 for four days, χ^2^ = 34.341 for six days, χ^2^ = 50.052 for eight days, *p* < 0.0001 for each time point).

**Figure 2:**
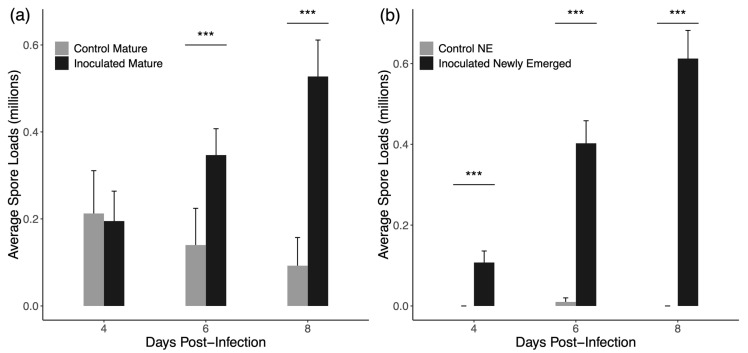
Average spore counts for drones +1 standard error. (**a**) Group inoculated mature drones show higher individual spore loads at 6 days and 8 days post-inoculation but not 4 days post-inoculation. (**b**) Group inoculated newly emerged drones have higher individual spore loads at all time points post-inoculation. *** denotes a significant difference.

Figure 2 shows the average spore loads observed in mature and newly emerged drones. In four-day cages of mature drones, control drones had more spores on average (212,000 ± 98,266 standard error) than the inoculated drones (150,000 ± 53,349 SE), but the median spore count for both groups was 0. Likewise, median spore counts for newly emerged drones in four-day cages was 0 for both inoculated and uninoculated drones. In every other case, inoculated drones presented with higher average and median spore count values. For mature drones, inoculated drones had higher median spore loads at six- and eight-days post-inoculation (Mann–Whitney U = 335.5 for six days and U = 287 for eight days, *p* < 0.001 for both) but not at 4 days post-inoculation (U = 739.5, *p* = 0.383). For newly emerged drones, inoculated drones presented with higher spore loads at every time point (U = 540 for four days, *p* = 0.0001; U = 254 for six days, *p* < 0.001; U = 160 for eight days, *p* < 0.001).

### 3.2. Drifting and Space Use

**Figure 3:**
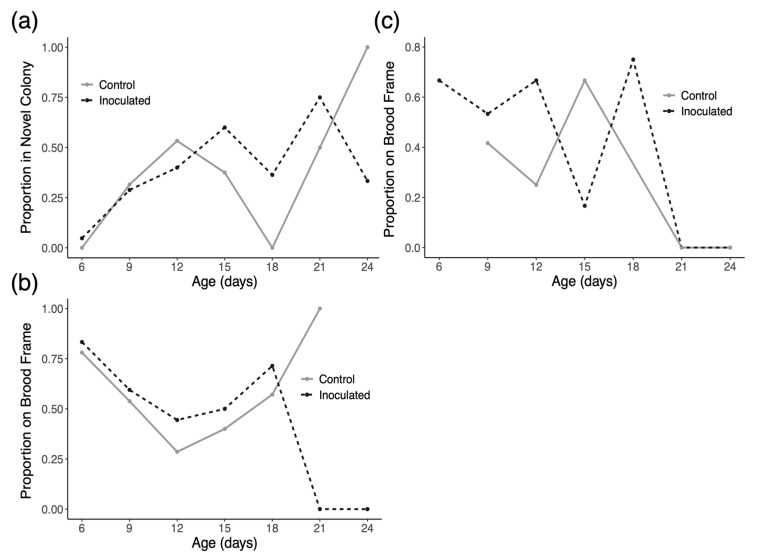
(**a**) Drifting rates of drones in trial 1 as a proportion of drones in each group observed in novel colonies at each inspection. Drifting increases over time but not due to infection. (**b**) Space use of drones in trial 1 as a proportion of drones in each group observed on brood frames in the original host colony at each inspection. Drones generally move towards outer portions of the nest as they age but infection has no evident effect. (**c**) Space use of drones in novel colonies in trial 1 as a proportion of drones in each group observed on brood frames in the novel colonies at each inspection.

**Figure 4:**
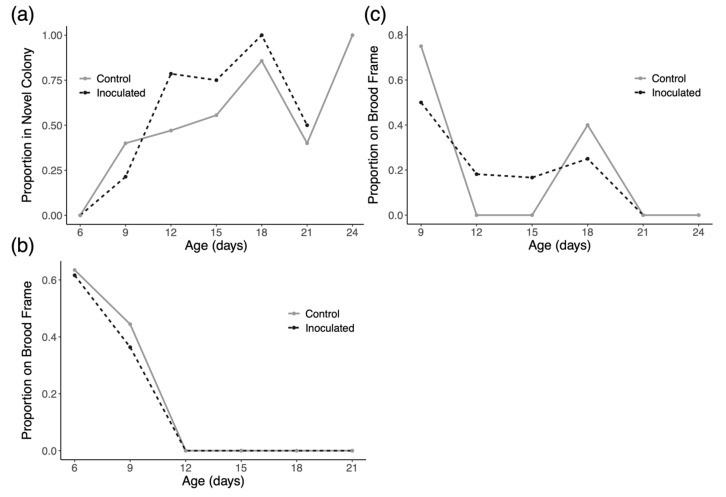
(**a**) Drifting rates of drones in trial 2 as a proportion of drones in each group observed in novel colonies at each inspection. Drifting increases over time but not due to infection. (**b**) Space use of drones in trial 2 as a proportion of drones in each group observed on brood frames in the original host colony at each inspection. Drones show strong movement towards outer portions of the nest as they age but infection has no evident effect. (**c**) Space use of drones in novel colonies in trial 2 as a proportion of drones in each group observed on brood frames in the novel colonies at each inspection.

**Figure 5:**
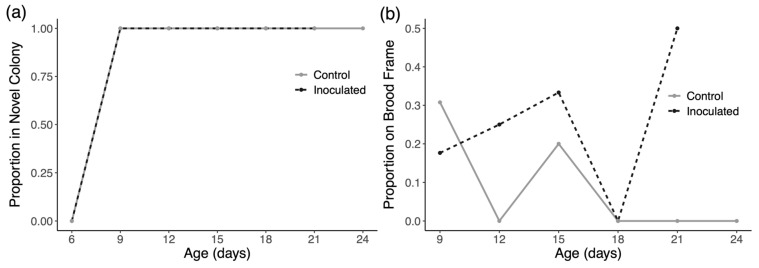
(**a**) Drifting rates of drones in trial 3 as a proportion of drones in each group observed in novel colonies at each inspection. In this trial, all drones drifted out of the original host colony by the second inspection and did not return. (**b**) Space use of drones in novel colonies in trial 3 as a proportion of drones in each group observed on brood frames in the novel colonies at each inspection.

In trial 1 of the experiment, we introduced 89 inoculated drones and 59 control drones and collected 158 and 112 observations of these marked drones, respectively. In trial 2 of the experiment, we introduced 91 inoculated drones and 90 control drones and collected 129 and 133 observations of these marked drones, respectively. In trial 3 of the experiment, we introduced 115 inoculated drones and 112 control drones and collected 88 and 86 observations of these marked drones, respectively. In trial 1, the model indicates that time has a significant positive effect on drifting in both groups (*p* < 0.0001), but there is no evidence of an effect of treatment on drift rates (Figure 3a). Similarly, for trial two, time has a significant positive effect on drifting in both groups (*p* < 0.0001), but there is no evidence of an effect of treatment on drift rates (Figure 4a). As shown in Figure 5a, all observed drones in both groups drifted to novel colonies by the second inspection and no marked drones were found in the host colony at any later inspection. We therefore performed no formal analysis for drifting in trial 3 and we cannot present any data from this trial concerning space use in the host colony during the trial.

Figure 3b and Figure 4b show the space use of drones in their original host colonies over time in trials one and two, respectively. Drones in trial 1 and 2 exhibited significantly decreased affinity for central portions of the nest over time in the host colony (*p* < 0.001 for trial one and *p* < 0.0001 in trial two). There was, however, no evidence of an effect of inoculation on how drones used space in their host colonies in either trial.

Looking at space use in the colonies that drones drifted into in each trial (Figure 3c, Figure 4c and Figure 5b), there appears to be no consistent pattern. Statistically, the only significant result is a negative effect of time on the usage of central nest areas in trial 2 only (*p* < 0.01).

### 3.3. Intruder Assay

**Figure 6:**
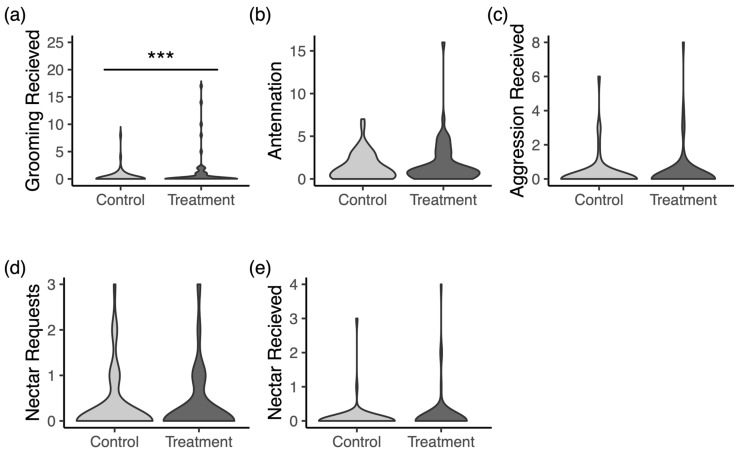
(**a**) Inoculated drones receive more grooming from putative guard bees in intruder assay arenas. (**b**–**e**) No differences were found for antennation, aggression received, nectar requests, or nectar received between inoculated and control drones. Counts of behaviors observed in scans of each trial are shown as violin plots. *** denotes a significant difference.

Across 4 rounds of trials, we recorded videos for 54 treatment drones and 61 control drones. The distributions of counts for each behavior from scan samples for both control and treatment drones are displayed in Figure 6. In the models for each behavior, only the reception of grooming behavior (Figure 6a) significantly differed between the groups. Inoculated drones were about three times more likely to receive grooming in the intruder arenas (*p* < 0.0001).

### 3.4. Social Interactions

**Figure 7:**
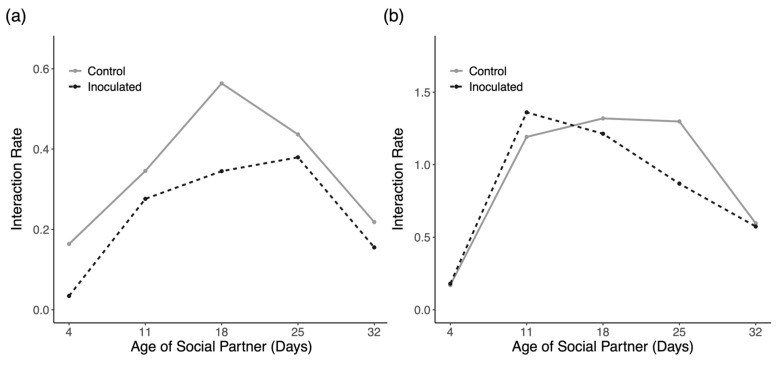
Interaction rates of each worker cohort with control and inoculated drones in (**a**) trial 1 and (**b**) trial 2.

Despite introducing the same number of marked workers and a similar number of drones in each trial of the experiment, we observed rather disparate interaction frequencies between the two trials. In trial one, we introduced 55 control drones and 58 inoculated drones and documented 134 and 107 interactions with each introduced group, respectively. In trial two, we introduced 47 control drones and 61 inoculated drones and documented 386 and 460 interactions with each introduced group, respectively. Figure 7 presents interaction rates with each particular cohort, which was calculated as the number of interactions with each cohort divided by the number of introduced drones in each group. This measure is visualized for general comparison and insight; however, there was no formal statistical test to perform for this data as there is only a single value for each cohort in each trial. Qualitatively, both trials show a consistent pattern in which older nurses, middle-aged bees, and young foragers interacted most frequently with foreign drones.

**Figure 8:**
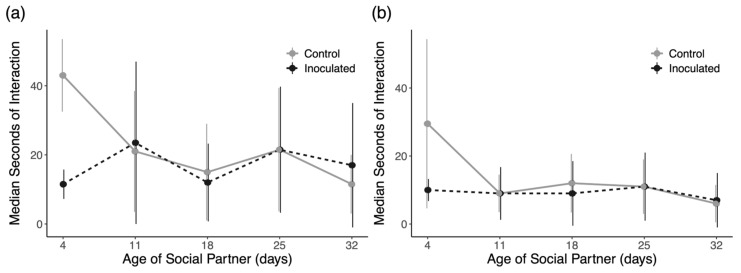
Duration of nectar exchanges with foreign drones by each cohort of workers in (**a**) trial 1 and (**b**) trial 2. Shown are the median durations with interquartile ranges.

**Figure 9:**
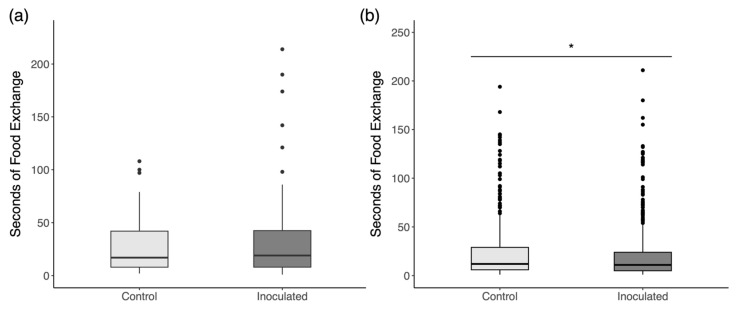
Duration of nectar exchanges with all workers, marked and unmarked, in (**a**) trial 1 and (**b**) trial 2. Workers in trial 2 show a slight reduction in nectar exchange duration with inoculated drones. Black dots are outlier data points. * denotes a significant difference.

Due to the very skewed distribution of duration data, we plotted the median values for interaction durations with each cohort with the interquartile range for each distribution in Figure 8. The results for each comparison of interaction durations with Mann–Whitney U tests show no significant differences for each cohort in both trials. We, therefore, see no evidence of an effect of inoculation on social interactions with any particular cohort. When data are pooled together, including interactions with unmarked workers of unknown age (Figure 9), there is a significant reduction in interactions with inoculated drones in trial 2 (Figure 9b; Mann–Whitney U = 95,884, *p* = 0.045) but not trial 1 (Figure 9a; U = 7025.5, *p* = 0.790).

### 3.5. Energetic Stress

**Figure 10:**
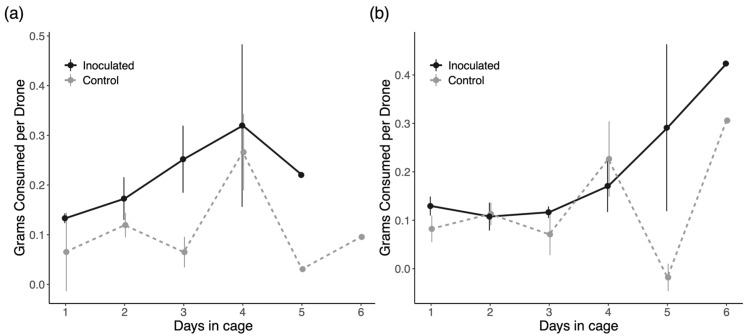
Average daily consumption of syrup by drones in cages in (**a**) trial 1 and (**b**) trial 2. Error bars represent ±1 standard error for each mean value. Missing error bars indicate data from a single remaining cage is plotted for that trial and group.

In trial 1 of this experiment, we gathered data on 40 inoculated drones in five cages and 32 control drones in four cages. In trial 2, we observed 44 treatment drones in six cages and 47 control drones in six cages. Average daily food consumption in each trial is shown in Figure 10. In trial 1, inoculation had a significant positive effect on food consumption (*p* = 0.007), but there is no evidence of an effect in trial 2 (*p* = 0.376). There is also a significant positive effect of time on food consumption for only day 4 in trial 1 (*p* = 0.002) and day 6 in trial 2 (*p* = 0.004), but this is independent of infection status. We saw no significant interactions between infection and time on feeding behavior.

## 4. Discussion

Across multiple experiments probing at the effects *N. ceranae* on inoculated drones, we find little evidence that infection causes behavioral changes that increase transmission of the pathogen. Nevertheless, the observed behavior of drones and workers, largely unaltered by infection, still has troubling implications for the ability of drones to serve as sources of pathogen spread both within and between colonies. We discuss these results with regard to the unique characteristics of drones, common social immunity defenses, and the limitations associated with each experiment.

### 4.1. Group Inoculation

Individual inoculation of bees is by far the most common method in *N. ceranae* research; however, group inoculation has been repeatedly verified as an effective method in workers [48,58,59,60]. We predicted that a modified group inoculation procedure using workers as vehicles for spore transmission would produce sufficiently consistent infections in drones. In comparison to group inoculation of workers, which has a success rate of 50% –100% seven days post-inoculation depending on worker age [48], we observed an overall rate of infection that is comparable at six and eight days post-inoculation in both newly emerged and mature drones (~75%). Importantly, these rates of infection were also significantly higher than the background rate of infection found in both newly emerged drones and mature drones at these time points (Figure 1).

Low rates of infection at 4 days post-inoculation is unsurprising as the life cycle of *N. ceranae* in cell culture models takes around 4 or 5 days [61]. A study of *N. ceranae’s* life cycle in actual honey bees, however, has shown complete production of new spores within 72 h [33]. While there may also be age- and sex-specific differences in the development of *Nosema* infection, the most likely reason we see low rates of infection at four days post-inoculation is because there is a delay between the introduction of the inoculate and feeding of the spores to drones. Higher rates of infection at six-and eight-days post-inoculation likely represent the further development of infections that exist at four-days post-inoculation but had not undergone a full spore formation cycle. Drones that were used for experiments after three- or four-days post-inoculation, such as in the feeding and drift experiment, very likely consumed spores during the 48 h inoculation period and were undergoing infection development over the course of these multiday experiments.

Consistent with previous reports, very few newly emerged bees presented with natural infections of *N. ceranae* [62]. In contrast, mature drones did exhibit a background rate of infection with a few control drones displaying very high spore loads (Figure 2). The rate of infection, the number of actually infected drones in each control and treatment group for each trial, is the primary consideration when interpreting later results; however, the presence of a few highly infected drones in control groups could also be impactful. The reduction in spore loads in mature control drones from 4-day cages to 6- and 8-day cages probably reflects the high mortality rate experienced by *N. ceranae* infected drones [63]. That is drones that were already infected prior to caging were unlikely to survive another six or eight days in the cage and did not make it to the sampling date or into later experiments.

Spore counts in our data are much lower than what has been reported in other studies where spore counts are commonly in the tens or hundreds of millions [32,60,64]. Drones have been shown to exhibit lower spore loads than workers when given the same infectious dose [63], but more importantly, most previous research has been performed with workers that are 12 days or more post-inoculation. Spore growth is exponential and so the difference between 8 and 12 days is likely massive and accounts for the differences in data presented here. Considering the lifespan of a honey bee, much of the potential transmission and important impacts of an infected bee almost certainly occur well before 12 days of infection. This is especially true for drones where it has been experimentally demonstrated that survival to 12 days post-infection is extremely low [63]. Despite aiming to replicate studies on workers, we found that drones in cages, whether inoculated or not, survive very poorly for long periods. Thus, for both relevance and practical purposes, shorter incubation periods were necessary when conducting experiments with drones.

### 4.2. Drifting and Space Use

Knowing that *N. ceranae* infection can affect the navigation and homing ability of workers [65,66], we hypothesized that infected drones may be more likely to drift than healthy drones. Results from this study confirm longstanding notions that drones drift at much higher rates than workers (~10–18% in reports on workers) and increase their tendency to drift as they age, but we do not observe any additional effect of *N. ceranae* infection on drifting behavior (Figure 3a, Figure 4a and Figure 5a). This finding agrees with similar studies that have also failed to directly link *N. ceranae* infection to increased drifting in workers [67,68]. One possibility is that homing success could be reduced in infected drones to the point that they drifted to far away colonies or simply never made it back to any of the colonies used in the experiment. Although our sample sizes did decrease greatly over the course of inspections, potentially denoting high mortality, we saw no substantial differences in the number of treatment and control drones at each inspection across trials. So, while it may be worthwhile to also test the homing abilities of *N. ceranae* infected drones, our results directly demonstrate rampant successful drifting by drones.

Drifting behavior has long been known to be promoted by common beekeeping practices while also being shaped by factors such as seasonality, inter-colony distances, and nectar availability [11,69]. Variation between our trials, including the stark results in trial 3, is probably attributable to differences in available landmarks at each particular field site or a general increase in drifting during later summer months in certain geographic areas [70]. One could argue that a more complex hive arrangement could have discouraged drift and in turn better reveal differences in how much treatment and control drones drift. However, our experimental design was intended to match conditions that are common to commercial apiary settings [71]. In this context, our results indicate that infected drones are absolutely capable of serving as a source of inter-colony disease transmission in most modern apiaries.

This experiment was also designed to address where drones spend time in the colonies they occupy. In general, drones exhibit a predictable pattern of space use as they age with younger drones preferring the brood area of the nest while older males prefer the periphery of the nest [72]. Given that *N. ceranae* infection has been shown to increase the affinity of workers for warmer, more central areas of the nest [39], we predicted that infected drones may spend more time in central brood areas in both their host colony and in novel colonies. Data from trials 1 and 2 (Figure 3b and Figure 4b) both show that drones display the same pattern of space use described in previous research in their home colonies, moving from central areas to the nest periphery over time. Although it is possible that movement towards the nest periphery could be due to aggression received by nestmates, we consider this possibility unlikely since both groups of drones showed this pattern and because we never witnessed aggressive encounters in our social interaction experiments. As described in earlier work, the spatial behavior of drones is likely driven by a transition from being predominately fed by nestmates to feeding directly from open nectar cells as they age [73,74]. In light of this, we find no support for the prediction that *N. ceranae* transmission is particularly enhanced towards central members of the nest due to prolonged affinity for the warmer brood area.

The spatial behavior of drones in novel colonies (Figure 3c and Figure 5b) is highly variable and difficult to predict. Similar to space use in host colonies, there was no observed effect due to infection itself and in one trial drones did significantly decrease their use of brood frames over time (Figure 4c). Ultimately, our inspections only present a snapshot of which drones were present in each colony and where they were located during that specific time of day. Drone movement throughout the day may be higher in unfamiliar colonies, but this would need to be tested in an experiment with more frequent sampling. Altogether, these results do not provide any clear indication that *Nosema ceranae* is actively manipulating the behavior of its drone hosts to enhance transmission.

### 4.3. Intruder Assay

While the previous experiment measured successful instances of drifting, it is possible that infected drones drift more often than healthy drones but are less successful at making it past the guard bees at the nest entrance. In the intruder assays performed in this study, we hypothesized that workers introduced to a foreign drone would resort to aggression as an adaptive response towards unrelated and potentially infected individuals that should not be allowed into the colony. Surprisingly, disinterest was by far the most common reaction to intruder drones in our assay arenas, which speaks to the ability of drones to successfully drift into novel colonies regardless of infectious status. Mortality was observed for both control and inoculated drones but was very rare. The only significant difference in how control and treatment drones were treated was a greater frequency of grooming inoculated drones (Figure 6). We therefore see that inoculated drones are not treated aggressively as intruders but are instead shown affiliative care behavior. The fact that infected drones received more grooming attention is a potential mechanism by which infection could be enhanced and should be the focus of future work.

Previous studies have shown similar results for honey bee workers inoculated with a viral pathogen in which infected workers received less aggression and more grooming and feedings than control workers as well as workers that were immunostimulated with bacteria but not infected [57]. Geffre et al. [57] linked these results to distinct changes in cuticular hydrocarbon profiles that may make infected bees uniquely able to avoid ejection and elicit sociable responses. We did not perform this additional analysis in our experiment, but it is likely that similar mechanisms are driving the reactions of workers to foreign drones infected with a novel parasite that is specifically known to suppress immune responses that elicit aggression towards sick individuals [45,75,76,77]. Other large differences in behaviors such as feeding and begging might have emerged if we used drones with further developed infections. However, mortality was high in many of our inoculation cages, and we decided to balance infection intensity with sufficient sample size. Combined with the drift experiment, we find solid evidence that favorable treatment may allow infected drones to enter novel colonies with great success.

### 4.4. Social Interactions

The ability of drones to transmit *N. ceranae* spores in a novel colony requires more than just the ability to make it past the colony entrance. Social interactions within a honey bee colony are nonrandom, and the consequences of passing on spores to individuals in a new colony fundamentally rely on which members choose to interact with infectious drones. We hypothesized that older members of the colony who are more disposable may be the most likely to engage in risky interactions with foreign sick drones, if drones were interacted with at all, and that interactions with infected drones should be shorter than those with putatively healthy drones [8,78]. Contrary to our predictions, inoculated drones received plenty of food from workers of all age groups (Figure 7), and there was very little evidence that workers cut off interactions with inoculated drones earlier than with control drones (Figure 8). Although there is a significant reduction in the time spent feeding inoculated drones by all observed workers collectively in trial 2 (Figure 9b), the effect size is very small (1 s difference) and likely irrelevant for actual transmission rates. By and large, both groups of foreign drones were treated similarly and in ways that are reasonably interpreted as affiliative.

Interaction rates with the oldest and youngest worker cohorts were low in both trials of the experiment. Low rates of interaction with the oldest foragers make sense as they are the least likely to be present in the colony in the middle of the day and those that are in the colony are likely near the entrance or edges of the nest [79]. Low rates of interaction with the youngest cohort of bees, while tempting to characterize as adaptive, may simply be due to these workers generally not being responsible for feeding nestmates in the way that nurses and middle-aged bees are [80,81,82]. We also observe that when drones do successfully solicit feeding from young bees, they tend to be fed for very long bouts which further suggests that young workers are not proactively isolating themselves from foreign drones.

It has been previously established that all workers feed drone bees native to their colony, although nurses are the most their common caretakers [73]. Our results indicate that older nurses and middle-aged bees play the main role in interacting with foreign drones, both sick and healthy. With more cohorts and better representation of ages within castes, we may have seen more interactions with nurses, in particular, but it is interesting to see more older workers (18–25 days) interacting with drones in this specific social immunity context. In terms of primary and secondary transmission events, our results suggest that infections could still be spread to many other colony members via nurses and middle-aged bees. Still, workers that actually received spores may change their future behavior in a way that limits pathogen transmission, such as through self-removal or precocious foraging [83].

Taken together, there is no evidence that workers avoid interactions with foreign drones and no evidence that workers actively discriminate between drones facing an immune challenge and drones that are presumably healthy. This experiment could be improved by individually identifying both drones and workers so that it could be determined if there are only a few workers feeding many drones as opposed to many different workers willingly engaging with foreign drones. Likewise, it would be useful to know if interactions are evenly spread across drones in each group or if certain individuals are monopolizing successful solicitation of feedings. Individual identification along with dissections after the trial to verify infection intensities and spore transfer would greatly enrich this experiment but would be laborious.

### 4.5. Energetic Stress

Due to the physical damage *Nosema* infection causes to the gut, we hypothesized that infected drones would consume more food than control drones as infections developed through the experiment. Previous work highlighted the refusal of sick workers to share food as a potentially adaptive response to limit spore transmission [38], but we did not test this as drones are already known to not feed any other members of the nest [73]. Across both of our trials, we obtained inconsistent results in support of our hypotheses (Figure 10). In conjunction with the intruder assay experiment, where there was no difference in food begging behavior between groups, we lack any strong evidence that increased hunger due to infection would strongly impact the transmission dynamics of *N. ceranae* by drones, in particular.

Clear differences in feeding behavior due to *Nosema* infection has been demonstrated in honey bee workers kept in cages with an attached feeder [37,84]. The inability for drones to learn how to use these kinds of feeders did make the experiment slightly more complicated and introduced notable issues. Part of the large amounts of food consumption seen in our study (decigrams per bee compared to micrograms in studies on workers) could be due to drones naturally eating more food than workers. However, the open comb also allowed drones to remove the solution in other ways that were witnessed such as covering their legs and other body parts with the sticky solution while clumsily walking over the comb. Future attempts at addressing this question could be improved with either a superior feeding apparatus or more sophisticated molecular methods characterizing actual metabolic dysfunction due to infection [85].

## 5. Conclusions

Although drones are known to carry various pathogens and parasites that have been implicated in unsustainable colony losses, we focused on *N. ceranae* because it is an important emerging pathogen of *A. mellifera* that is tractable, well-studied, and thought to be disproportionately transmitted by drones. The results presented here largely indicate that western honey bee colonies are incapable of recognizing males infected with the parasite and are, therefore, unable to mount competent social immunity defenses against this novel pathogen. The scant evidence of direct manipulation of host behavior is unfortunately no consolation as the natural behavior of drones is already highly conducive to inter-colony pathogen transmission. Drones do not feed themselves, for example, and spend a great deal of time moving about the nest begging for food and engaging in trophallaxis. We did find a great deal of variability between trials in several experiments suggesting that the nature of drifting, and pathogen spread, may be stochastic and or based on variables unexplored in this work. Future work should also attempt to work with larger numbers of colonies. For managed colonies living in environments affected by a web of interconnected issues, it is likely that *N. ceranae* infections are a common additional problem interacting with other stressors to devastate colonies.

## Data Availability

The original contributions presented in this study are included in the article. Further inquiries can be directed to the corresponding author.
